# Gene Silencing of Toll-Like Receptor 2 Inhibits Proliferation of Human Liver Cancer Cells and Secretion of Inflammatory Cytokines

**DOI:** 10.1371/journal.pone.0038890

**Published:** 2012-07-16

**Authors:** Yuzheng Huang, Bing Cai, Ming Xu, Zhiqin Qiu, Yonghui Tao, Ying Zhang, Jie Wang, Yongliang Xu, Yonghua Zhou, Jing Yang, Xiaofeng Han, Qi Gao

**Affiliations:** 1 Key Laboratory on Technology for Parasitic Disease Prevention and Control, Ministry of Health, Jiangsu Institute of Parasitic Diseases, Wuxi, Jiangsu, China; 2 Wuxi People’s Hospital of Nanjing Medical University, Wuxi, Jiangsu, China; 3 Wuxi Secondly People’s Hospital of Nanjing Medical University, Wuxi, Jiangsu, China; National Taiwan University Hospital, Taiwan

## Abstract

**Background:**

Toll-like receptors (TLRs) are key factors in the innate immune system and initiate the inflammatory response to foreign pathogens such as bacteria, fungi and viruses. In the microenvironment of tumorigenesis, TLRs can promote inflammation and cell survival. Toll-like receptor 2/6 (TLR2/6) signaling in tumor cells is regarded as one of the mechanisms of chronic inflammation but it can also mediate tumor cell immune escape and tumor progression. However, the expression of TLR2 and its biological function in the development and progression of hepatocarcinoma have not been investigated. This study aimed to determine the expression of TLRs 1–10 in the established human hepatocellular carcinoma cell line BLE-7402, to investigate the biological effect of TLR2 on cell growth and survival.

**Methods:**

TLR expression in BLE-7402 cells was assayed by RT-PCR, real-time PCR and flow cytometry (FCM). To further investigate the function of TLR2 in hepatocarcinoma growth, BLE-7402 cells were transfected with recombinant plasmids expressing one of three forms of TLR2 siRNA (sh-TLR2 RNAi(A, B and C)). TLR2 knockdown was confirmed using RT-PCR, real-time PCR and fluorescence microscopy. Tumor cell proliferation was monitored by MTT assay and secreted cytokines in the supernatant of transfected cells were measured by bead-based FCM, the function of TLR2 siRNA was also investigated in vivo.

**Results:**

The BLE-7402 cell line expressed TLRs 2 to 10 at both mRNA and protein levels. TLR2 was the most highly expressed TLR. While all the three siRNAs inhibited TLR2 mRNA and protein expression, sh-TLR2 RNAi(B) had the strongest knockdown effect. TLR2 knockdown with sh-TLR2 RNAi(B) reduced cell proliferation. Furthermore, secretion of IL-6 and IL-8 was also reduced. The result showed a drastic reduction in tumor volume in mice treated with sh-TLR2 RNAi(B).

**Discussion:**

These results suggest that TLR2 knockdown inhibit proliferation of cultured hepatocarcinoma cells and decrease the secretion of cytokines. It is suggested that TLR2 silencing may worth further investigations for siRNA based gene therapy in treatment of hepatocarcinoma.

## Introduction

Hepatocellular carcinoma or liver cancer is considered to be a primary cancer originating from liver cells; it is one of the most devastating cancer form, especially in China. Currently, lacking of effective treatment lead for searching novel treatment strategy, such as gene therapies. Short interfering RNA, siRNA may be offered as an novel therapy once a good target is found. It is recently suggested TLRs are expressed in many human tumors [1],

Toll-like receptors (TLRs) are a highly conserved family of type I transmembrane receptors that recognize specific pathogen-associated molecular patterns (PAMPs), e.g. lipopolysaccharide, lipotechoic acid and other bacterial wall components [1], [2], and it can also mediate tumor cell immune escape and tumor progression. Human TLRs have a cytoplasmic domain which is homologous to the cytoplasmic domain of the human interleukin (IL)-1 receptor [3]. To date, 11 mammalian TLRs have been identified and characterized.

Recently, new research has revealed that TLRs are expressed by many human tumors [2], [3], [4], [5], [6],including prostate cancer, lung cancer, breast cancer and hepatocellular carcinoma. Although the TLRs have different functions in different tumor cells, some results have indicated that TLR signaling can play a role in tumor growth and progression. For example, TLR2 signaling can promote lung cancer cell growth and resistance to apoptosis [7], [8]; TLR3-dependent signaling can directly lead to apoptosis in human breast cancer [6]; through their actions on metalloproteases and integrins, TLR2 and TLR9 can lead to increased invasiveness and metastasis [8], [9]; TLR4 can mediate metastasis that actively advances tumor cell invasion, proliferation, and survival of prostate cancer cells [10].

Toll-like receptor 2/6 (TLR2/6) signaling in tumor cells is of particular interest as it is regarded as one of the mechanisms of chronic inflammation but it can also mediate tumor cell immune escape and tumor progression. Additionally, TLR2 act as a potential antiviral mechanism in hepatitis B-infected hepatocyte cell lines [11].

TLRs are expressed on a wide variety of tumor cells and are suspected to play important roles in the initiation and progression of cancer, however the expression of TLRs by hepatocarcinoma cells has not been examined in a systematic manner and little is known about TLR interaction with disease progression. In this study, we aimed to determine the expression of TLRs 1–10 in the established human hepatocellular carcinoma cell line BLE-7402. We additionally aimed to investigate the biological effect of TLR2 on cell growth and survival, and to assess its potential in the field of cancer therapy.

## Materials and Methods

All experiments complied with the current laws of China.

### Cell Line

The human hepatocellular carcinoma cell line BEL-7402 was purchased from the cell bank of the Chinese Academy of Sciences (Shanghai, China). BEL-7402 was grown without antibiotics in 5% CO_2_ at 37°C in RPMI-1640 (Gibco, Invitrogen, Carlsbad, CA) containing 10% FBS.

### Construction of siRNA-expressing Plasmids

Three small interfering oligonucleotides (A: 5′-aactatccactggtgaaacaa-3′, B: 5′- aaacttgtcagtggccagaaa-3′, C: 5′- aaagtcttgattgattggcca-3′) were designed based on the sequences of human TLR 1–10 (Table 1) and synthesized. RNAi plasmid vectors (Figure S1) expressing siRNAs for TLR2 under the control of a human CMV promoter, were generated by inserting pairs of the annealed DNA oligonucleotides specific for TLR2 at the HindIII and BamHI sites sequentially into the pGenesil-1 plasmid (shTLR2, Table 2). A pGenesil-1-scrambled vector was used as a control.

**Table 1:** PCR primers of human TLRs.

Genes	Primer (5′-3′)	Amplification size(bp)
TLR1	For: AGATTTCTTGCCACCCTACTG	122 bp
	Rev: GCTCAACCCCAGAAATTTTAG	
TLR2	For: AACTTACTGGGAAATCCTTAC	264 bp
	Rev: AAAAATCTCCAGCAGTAAAAT	
TLR3	For: GCATTTGTTTTCTCACTCTTT	131 bp
	Rev: TTAGCCACTGAAAAGAAAAAT	
TLR4	For: CGAGGAAGAGAAGACACCAGT	106 bp
	Rev: CATCATCCTCACTGCTTCTGT	
TLR5	For: AGCTTCAACTATATCAGGACA	383 bp
	Rev: TGGTTGGAGGAAAAATCTAT	
TLR6	For: CTTCCATTTTGTTTGCCTTAT	123 bp
	Rev: AGCGGTAGGTCTTTTGGAAC	
TLR7	For: AAACTCCTTGGGGCTAGATG	149 bp
	Rev: AGGGTGAGGTTCGTGGTGTT	
TLR8	For: CTGTGAGTTATGCGCCGAAGA	246 bp
	Rev: TGGTGCTGTACATTGGGGTTG	
TLR9	For: CGCCCTGCACCCGCTGTCTCT	168 bp
	Rev: CGGGGTGCTGCCATGGAGAAG	
TLR10	For: AGAAGAAAGGGAACTGATGAC	279 bp
	Rev: CCTGCCAGTAAATACCAAGT	
GAPDH	For: GGATTTGGTCGTATTGGG	205 bp
	Rev: GGAAGATGGTGATGGGATT	

### Qualitative RT-PCR

TRIzol reagent (Invitrogen) was used to isolate total RNA according to the manufacturer’s instructions. A 4 µg aliquot of total RNA was mixed with an oligo-dT primer and myeloblastosis virus (MLV) reverse transcriptase (Promega, Madison, WI). PCR primers for TLR 1–10, together with GAPDH as control, were designed as shown in Table 1. The PCR conditions were as follows: 95°C for 5 min, followed by 30 cycles of 95°C for 1 min, 55°C for 1 min, and 72°C for 1 min. The final extension was at 72°C for 10 min. PCR products were analyzed on 1.5% (wt/vol) agarose gels containing 0. 5 µg/mL ethidium bromide and were visualized under UV light. Band density was analyzed and quantified using ImageQuant software (Molecular Dynamics, Sunnyvale, CA).

### Real-time PCR

The reaction mixture contained 45 µL DEPC-H_2_O, 1.0 µL cDNA at a 1∶100 dilution, 2.0 µL (10 µM) of each primer and freeze-dried powder according the manfacturer’s instructions of the AccuPower Greenstar® qPCR premix. The procedure for PCR was as follows: 95°C for 5 min, followed by 40 cycles of 94°C for 30 sec, 55°C for 30 sec, 72°C for 30 sec. Fluorescence was measured after each extension step of 72°C for 30 sec (Eppendorf, Germany). At the end of the reaction, the melting curve of each sample was analyzed. BIONEER Exicycler™ analysis software (Bioneer Corp., Daejeon, Korea) was used to obtain the C_t_ values. Relative expression of the target genes was determined by the 2^−ΔΔ CT^ method [12].

### Flow Cytometry Detection of TLR Protein Expression

A suspension of 2×10^6^ cultured BEL-7402 cells was prepared and permeabilized to detect TLR protein expression. The cells were collected and washed twice with 1×PBS, then stained with 5 µL purified anti-human TLR2 antibody (Santa Cruz Biotechnology, Santa Cruz, CA) at 4°C for 1 h protected from light. After washing twice with 1×PBS, the cells were collected by low speed centrifugation (1500 g, 10 min) and incubated with 2 µL PE-conjugated goat anti-rabbit IgG mAb (Santa Cruz Biotechnology) at 4°C for 30 min in the dark, followed by two washes with 1×PBS. Finally, the stained cells were suspended in 500 µL 1×PBS, and analyzed by flow cytometry (FACScalibur; Becton Dickinson, Franklin Lakes, NJ) using BD CellQuest software. The negative control was processed in the same way but without the anti-TLR2 antibody.

### RNA Interference Assay

Cells were transfected with the GFP-expressing plasmid pGsil-1 (Genesil, Wuhan, China) containing siRNA directed against TLR2, as shown in Table 2. The transfected cells were seeded at 2×10^5^ cells/well into 6-well dishes and cultured overnight until they reached 70% confluence. Transfections were performed using Lipofectamine™ 2000 reagent (Invitrogen) following the manufacturer’s instructions. Four µg of plasmid DNA (sh-TLR2 RNAi(A), sh-TLR2 RNAi(B), sh-TLR2 RNAi(C), vector pGenesil-1 and scrambled siRNA) were used for transfection into cells respectively (SunShineclean™ Without-Endotoxin Plasmid Mini Extraction Kit, SunShineBio, China). Transfected cells were incubated for 72 h, then fluorescence was observed using a fluorescence microscope. At this time cells were also collected for FCM and immunofluorescence assay.

**Table 2:** Sequences of siRNA against TLR2.

Name of siRNA	TLR2 sequences(5′-3′)
TLR2A	aactatccactggtgaaacaa
TLR2B	aaacttgtcagtggccagaaa
TLR2C	aaagtcttgattgattggcca

### Immunofluorescence Analysis

BEL-7402 cells were transfected with various siRNA plasmids and cultured overnight, then fixed with alcohol for 30 min and blocked in 1×PBS (pH 7.4) solution with 3% BSA. The anti-TLR2 antibody (sc-166900, Santa Cruz Biotechnology) was added at a 1∶200 dilution and incubated overnight at 4°C in a humidified box. After washing, the fluorescent secondary antibody (sc-22766, Santa Cruz Biotechnology) was added at a 1∶100 dilution and incubated for 2 h. The cells were then washed three times with 1×PBS, and counter-stained with DAPI. Confocal microscopy was performed and fluorescence was analyzed using a fluorescence microscope (Leica DMI4000B, Buffalo Grove, IL).

### Cell Proliferation Assay

The MTT assay (Sigma, St. Louis, MO) was used to evaluate cell proliferation after transfection. Cells were cultured in 96-well cell plates (1×10^4^/well, 5 wells per condition) overnight, and cell transfections were performed according to the manufacturer’s instructions. After 48 h, a sample of the transfected cells was collected as the 0 h sample, while the other cells continued in culture for 24 h, 48 h, and 72 h. At the end of each treatment period, MTT was added to the culture medium in each well at a concentration of 5 mg/mL (Sigma Chemical Co., St Louis, MO), and then incubated for 4 h at 37°C. The supernatant was then removed and cells were mixed with 100 µL/well dimethyl sulfoxide. The absorption was measured using an automatic microplate spectrophotometer (340st; Anthos Zenyth, Salzburg, Austria) at 490 nm.

### Human Inflammatory Cytokine Assay

Supernatant was collected from transfected cells. IL-6 and IL-8 levels were detected using the human inflammatory cytokine kit (BD™ Cytometric Bead Array) according to the manufacturer’s instructions.

### Experiment of Mouse Model in vivo

Athymic male nude mice (Balb/c -nu/nu; 5–7 weeks of age) were divided in to five treatment groups with ten mice per treatment group. Animal handling and experimental procedures were approved by the Animal Experiments Committee of Nanjing Medical University. A suspension of BEL-7402 cells (1×10^7^) in 500 µl of PBS was injected into subcutaneous of abodomen. This cell concentration was necessary to achieve consistent local tumor growth within 10 days of implantation. At days 10 and 15 post-implantation, the tumors were injected respectively with plasmid DNA (sh-TLR2 RNAi(A), sh-TLR2 RNAi(B), sh-TLR2 RNAi(C), vector pGenesil-1 and scrambled siRNA),with 150 µg each time. The tumor scale was determined by visual inspection and measured by vernier caliper 15 d after the second injection.

### Statistical Analysis

Data shown are from at least three separate experiments and are represented as the means ± standard deviation (SD). Student’s *t*-test and ANOVA were used to determine significance, and differences were considered significant at a *P* value of less than 0.05.

## Results

### Expression of TLRs in the BEL-7402 Cell Line

**Figure 1:**
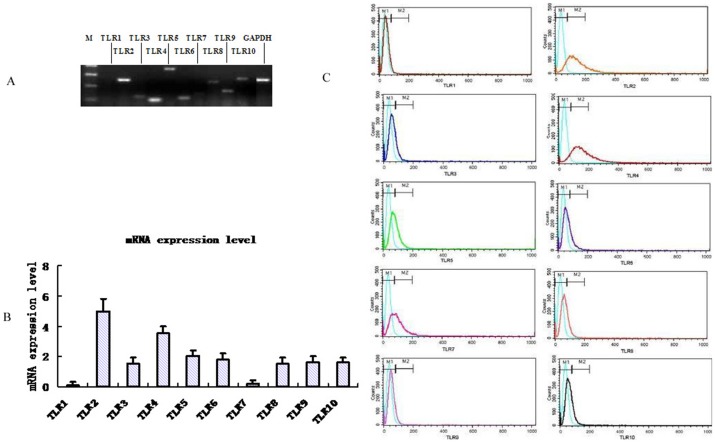
mRNA and protein expression levels of TLRs (TLR1–10) in human liver cancer cell line BEL-7402. Semi-quantitative RT-PCR result of TLRs in BEL-7402. GAPDH mRNA was used as the control(A). Real-time RT-PCR of TLRs in BEL-7402. The expression of TLR7 was normalized to 1.0 as its level was lowest among all TLRs(B). TLR protein expression levels in BEL-7402 by flow cytometry(C). All results are representative of three separate experiments.

Semi-quantitative RT-PCR analysis revealed that mRNA for TLRs (TLR 2–10) is expressed in BEL-7402 cells (Figure 1A), while TLR1,7 mRNA expression was barely detectable. Further analysis by real-time PCR showed that TLR7 is expressed at the lowest level. We therefore chose to refer all other TLR mRNA levels relative to the level of TLR7 mRNA. TLR2 and TLR4 showed the highest expression levels, which may indicate they have some function in BEL-7402 cell growth and progression (Figure 1B).

TLR protein expression levels were determined by flow cytometry (FCM). TLR2 was expressed at highest levels of TLR1–10, the other TLRs were all expressed at moderate levels expressed or weakly (Figure 1C). Taken together, our results demonstrate that BEL-7402 cells in culture express TLR2–TLR10, with TLR2 being the most highly expressed. This result encouraged us to further investigate the function of TLR2 on the growth and progression of BEL-7402 cells.

### Efficient Knockdown of TLR2 Expression in the BEL-7402 Cell Line by Three siRNAs

**Figure 2:**
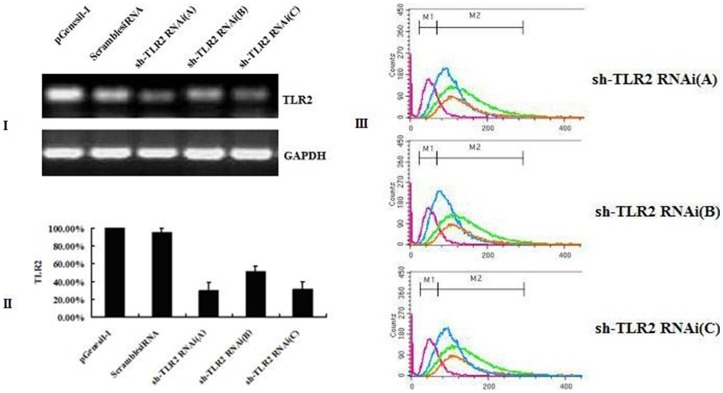
Transfection and silencing of TLR2 expression after siRNAs in BEL-7402. I, RT-PCR of TLR2 from pGenesil-1 vector, ScrambledsiRNA, sh-TLR2 RNAi(A, B, C) transfected BEL-7402. II, expression of TLR2 at mRNA level in pGenesil-1 vector, ScrambledsiRNA, and sh-TLR2 RNAi(A, B, C) transfected BEL-7402 with real-time PCR. III, flow cytometry analysis of TLR2 expression, student’s *t*-test and ANOVA were used to determine significance.

To further investigate the potential function of TLR2 in hepatocarcinoma, we used small hairpin RNAs to knockdown endogenous TLR2 gene expression [Gene ID: 10333]. Plasmid vectors were constructed to express three different siRNAs, sh-TLR2 RNAi(A), sh-TLR2 RNAi(B), sh-TLR2 RNAi(C). All three experimental siRNA vectors were transfected into BEL-7402 cells respectively, with scrambled siRNA vector and the empty vector as controls. After 48 h, the average transfection efficiency was about 70% as estimated by green fluorescence positive cells under the florescence microscope. All three experimental siRNAs effectively reduced TLR2 mRNA expression in transfected cells (Figure 2I). TLR2 mRNA levels were 29.85% ±9.2%, 50.75±6.7% and 31.34±8.3% of the empty vector control with sh-TLR2 RNAi(A), sh-TLR2 RNAi(B) and sh-TLR2 RNAi(C) respectively, a statistically significant decrease in expression compared to the empty vector control cells (*P*<0.05) (Figure 2II). No statistically significant decrease in expression was seen in cells transfected with the scrambled siRNA (*P>*0.05). TLR2 protein expression was reduced by all three siRNAs with levels of 33.0% ±3.0% (sh-TLR2 RNAi(A)), 57.9% ±4.7% (sh-TLR2 RNAi(B)) and 43.7% ±4.5% (sh-TLR2 RNAi(C)) of the empty vector control (Figure 2III). No significant difference in TLR2 protein expression was seen in the scrambled siRNA control (*P>*0.05). These data indicate that sh-TLR2 RNAi(B) is the most efficient recombinant plasmid in silencing TLR2. We therefore selected to use only sh-TLR2 RNAi(B) in further experimentation.

**Figure 3:**
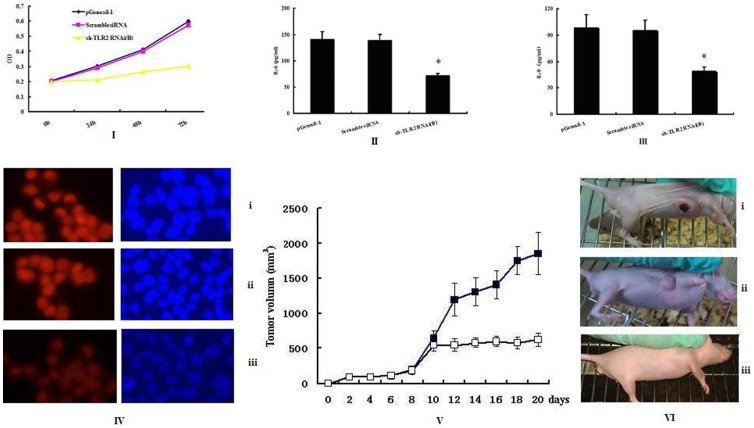
TLR2 expression and functional effect after TLR2 knockdown in BEL-7402 cell and in vivo. I, MTT analysis of the proliferative rate of pGenesil-1 vector, ScrambledsiRNA and sh-TLR2 RNAi(B) transfected cells. II and III, IL-6 and IL-8 presence in the supernatant secreted by pGenesil-1 vector, Scrambled siRNA and sh-TLR2 RNAi(B) transfected cells. Cell supernatant was collected and analyzed using flow cytometry. All results are representative of three separate experiments, student’s *t*-test and ANOVA were used to determine significance. IV, immunofluorescence analysis of gene-specific siRNA on TLR2 protein expression in pGenesil-1 vector(i), Scrambled siRNA(ii) and sh-TLR2 RNAi(B) (iii) transfected cells. Nuclear staining performed using DAPI (blue) (200×). TLR2 silencing suppresses tumor growth in an mouse model. Comparison of volume of tumors from each treatment group was shown. Treatment with BEL-7402 cell (VI_i), sh-TLR2 RNAi(B) constructive DNA was injected in tumor group(VI_ii), Significant differences from control groups(V.▪, VI_iii) were represented by asterisks (*P*<0.05).

TLR2 protein expression in BEL-7402 cells was further assayed by immunostaining with an anti-TLR2 antibody and visualized under a fluorescence microscope. Positive staining was dramatically reduced by sh-TLR2 RNAi(B) compared to the empty vector control (Fig. 3IV). No significant difference was seen in the staining levels of scrambled siRNA control compared to the empty vector control (Fig. 3IV).

Thus, we have demonstrated specific siRNA-directed knockdown of the TLR2 gene in the human hepatocellular carcinoma cell line BEL-7402, and shown that sh-TLR2 RNAi(B) was the most efficient recombinant plasmid in silencing TLR2.

### Proliferation of the Transfected BEL-7402 Cell Line Following TLR2 Gene Knock Down

We used the MTT assay to determine the effects of sh-TLR2 RNAi(B)-mediated TLR2 silencing on cell proliferation. Cells were cultured for 0 h, 24 h, 48 h, and 72 h after 48 h of transfection. The percentage of viable cells was reduced in the presence of TLR2 siRNA by ∼50% on average as compared with cells without any treatment. The proliferative ability of BEL-7402 cells was reduced by sh-TLR2 RNAi(B) transfection (Figure 3I). No significant difference was observed in the cells transfected with the siRNA control (*P>*0.05).

### Down-regulation of TLR2 by siRNA Blocks its Downstream Signaling in BEL-7402 Cells

The biological changes caused by TLR2 down-regulation may be due to alteration of TLR2-mediated downstream signaling leading to changes in subsequent functions.

TLR2 may play a role in TLR2/MyD88 signaling and subsequent downstream functions [17], and we presume that cytokines secretion is an outcome of TLR2 signaling. Therefore, we designed a protocol to examine the level of inflammatory cytokines in BEL-7402 cells following TLR2 gene knockdown. BEL-7402 cells were transfected with sh-TLR2 RNAi(B). After 72 h supernatant was removed and the levels of IL-6 and IL-8 determined by cytometric bead assay. IL-6 and IL-8 levels were found to be markedly depressed. IL-6 was reduced by 50.2±2.6% and IL-8 by 49.7±3.5% when compared with empty vector controls (*P*<0.05); no significant difference was seen in siRNA-transfected controls (Figure 3II and Figure 3III).

### TLR2 Gene by siRNA Inhibits Tumorigenesis in Nude Mice

By having established the significant effects of TLR2 knockdown by RNAi in BEL-7402 cells *in vitro*, we wonder whether TLR2 RNAi would also suppress the tumorigenicity of BEL-7402 tumors in nude mice. On days 7 and 14 post-implantation, the tumors were injected with plasmid DNA expressing TLR2 siRNA. The gross morphology of primary tumors were examined. Tumors were measured and a drastic reduction in tumor volume was observed in mice treated with sh-TLR2 RNAi(B) (Figure 3V–VI ii).

## Discussion

Toll-like receptors (TLRs) are a conserved family of receptors that sense the presence of pathogens by recognizing PAMPs [13], [14]. In addition they are also involved in regulating inflammation [15], a role which has been suggested to be at least partially dependent on the ability of TLRs to recognize several endogenous TLR ligands known as damage-associated molecular patterns (DAMPs). Recently, many studies on TLRs and their potential role in cancer research and therapy have been reported. Yang et al [16] investigated the potential roles of TLR2 signaling on tumor metastasis in a mouse model of intravenously injected B16 melanoma cells. The result demonstrate that TLR2 is an attractive target against metastasis, anti-TLR2 antibody could be used to combate the life-threatening metastasis.Thompson et al [11] showed that hepatoma cell lines express functional TLR2 receptors, which, when stimulated, initiate a signaling cascade that inhibits hepatitis B virus. Xu et al [17] detected peripheral blood mononuclear cells from HBV infected patients showed decreased levels of TLR7 expression and TLR9 mRNA, but an increase in TLR9 expression at the protein level.

From these studies, we know that TLR2, TLR7 and TLR9 play key roles in liver diseases and TLR2 has an potential function in the regulation of tumor tolerance, cancer progression and metastasis [13]. Orihara et al [18] have revealed that TLR2 and/or TLR4 on circulating monocytes are significantly up-regulated in bacterial infection. TLR2 expression levels on monocytes has been shown to provide critical information when planning treatment against bacterial infectious diseases [18] and filarial diseases [19], ovarian cancer [20], liver diseases [21], and bacterial infectious disease [22], [23], [24], [25]. However, the growth, proliferation and metastasis of hepatocellular carcinoma are complicated and dynamic processes. Each process is likely to be associated with the actions (and interplay) of several TLRs [26]. In the light of this, we decided to explore the expression of other TLRs in hepatocellular carcinoma and investigate whether these could affect the growth, progression and survival of liver cancer. Our experiments demonstrate that TLR 2–10 are expressed in BEL-7402 cells at both the mRNA and protein levels. We have shown, by real-time PCR analysis and flow cytometry, that TLR2 is the most highly expressed of the TLRs.

There is emerging evidence that many different cell types in the liver express TLR2 [27]. such as Kupffer cells, hepatocytes and biliary epithelial cells. TLR-mediated signals result in hepatitis B, hepatitis C, alcoholic liver disease, non-alcoholic liver diseases, primary biliary cirrhosis, primary sclerosing cholangitis, hepatic fibrosis, ischemic-reperfusion injury and liver allograft rejection [27]. Huang et al. showed that *Listeria monocytogenes* activated mitogen-activated protein kinases and nuclear factor-κB in tumor cells through TLR2 signaling, resulting in the increased production of nitric oxide and interleukin-6 and increased proliferation of tumor cells [7]. Kim et al. showed that Lewis lung carcinoma (LLC) was the most powerful macrophage activator leading to production of interleukin-6 and tumor-necrosis factor-α through activation of the Toll-like receptor (TLR) family members TLR2 and TLR6 [9] and their results explained how advanced cancer cells change the host innate immune system and thus generate an inflammatory microenvironment. Huang et al. showed that the microenvironment of large tumors favors bacterial survival, which in turn directly accelerates tumor growth by activating tumor cell Toll-like receptor 2 [7].

Proinflammatory factors such as nitric oxide, IL-6 and IL-12 were shown to be released from tumor cells in previous studies [10]. Both IL-6 and IL-12 are known to help tumor cells resist cytotoxic T lymphocytes and natural killer cells, leading to evasion from immune surveillance [10]. In our study, TLR2 was selected to explore the function of TLRs in the growth of BEL-7402 cells. Our study confirms the importance of TLR2-mediated tumor proliferation, analysis of inflammatory cytokines demonstrated depressed production of TLR2 expression after TLR2 knockdown, and showed that the ability of BEL-7402 cells to resist immune cell attack and facilitate evasion from immune surveillance could be decreased. This suggests that all of the phenotypic changes observed in these cells were mediated by suppressing the action of TLR2.

The mouse mode of cancer was also investigated, and the volume of tumor from siRNA treatment was shown. By having established the significant effects of TLR2 knockdown by RNAi in BEL-7402 cells in vitro, we wonder whether TLR2 RNAi would also suppress the tumorigenicity of BEL-7402 tumors in nude mice. On days 10 and 15 post-implantation, the tumors were injected with expressing plasmid DNA sh-TLR2 RNAi. The gross morphology of primary tumors were examined. A drastic reduction in tumor volume was observed in mice treated with sh-TLR2 RNAi(B).

The results presented in this paper provide evidence that TLR2 knockdown not only in vitro but in vivo could inhibit the growth of liver tumors. TLR2-mediated cancer growth appears to be an important factor in tumor progression. The use of systemically delivered TLR2 siRNA may thus provide a novel treatment for the prevention of cancer progression leading to better prospects of survival.

## Supporting Information

Figure S1 **RNAi plasmid vectors (pGenesil-1 plasmid).**
(JPG)

## References

[1] Muccioli M, Sprague L, Nandigam H, Pate M, Benencia F (2012). Toll-like receptors as novel therapeutic targets for ovarian cancer.. ISRN Oncol.

[2] Hassan F, Islam S, Tumurkhuu G, Naiki Y, Koide N (2006). Intracellular expression of toll-like receptor 4 in neuroblastoma cells and their unresponsiveness to lipopolysaccharide.. BMC cancer.

[3] Ilvesaro JM, Merrell MA, Swain TM, Davidson J, Zayzafoon M (2007). Toll like receptor-9 agonists stimulate prostate cancer invasion in vitro.. Prostate.

[4] Droemann D, Albrecht D, Gerdes J, Ulmer AJ, Branscheid D (2005). Human lung cancer cells express functionally active Toll-like receptor 9.. Respir Res.

[5] Molteni M, Marabella D, Orlandi C, Rossetti C (2006). Melanoma cell lines are responsive in vitro to lipopolysaccharide and express TLR-4.. Cancer Lett.

[6] Salaun B, Coste I, Rissoan MC, Lebecque SJ, Renno T (2006). TLR3 can directly trigger apoptosis in human cancer cells.. J Immunol.

[7] Huang B, Zhao J, Shen S, Li H, He KL (2007). Listeria monocytogenes promotes tumor growth via tumor cell toll-like receptor 2 signaling.. Cancer Res.

[8] Ren T, Wen ZK, Liu ZM, Liang YJ, Guo ZL (2007). Functional expression of TLR9 is associated to the metastatic potential of human lung cancer cell: functional active role of TLR9 on tumor metastasis.. Cancer Biol Ther.

[9] Kim S, Takahashi H, Lin WW, Descargues P, Grivennikov S (2009). Carcinoma-produced factors activate myeloid cells through TLR2 to stimulate metastasis.. Nature.

[10] Hua D, Liu MY, Cheng ZD, Qin XJ, Zhang HM (2009). Small interfering RNA-directed targeting of Toll-like receptor 4 inhibits human prostate cancer cell invasion, survival, and tumorigenicity.. Mol Immunol.

[11] Thompson AJ, Colledge D, Rodgers S, Wilson R, Revill P (2009). Stimulation of the interleukin-1 receptor and Toll-like receptor 2 inhibits hepatitis B virus replication in hepatoma cell lines in vitro.. Antivir Ther.

[12] Livak KJ, Schmittgen TD (2001). Analysis of relative gene expression data using real-time quantitative PCR and the 2(-Delta Delta C(T)) Method.. Methods.

[13] Huang B, Zhao J, Unkeless JC, Feng ZH, Xiong H (2008). TLR signaling by tumor and immune cells: a double-edged sword.. Oncogene.

[14] Kawai T, Akira S (2010). The role of pattern-recognition receptors in innate immunity: update on Toll-like receptors.. Nat Immunol.

[15] Huang Y, Yang G, Kurian D, Xu M, Dai Y (2011). Proteomic patterns as biomarkers for the early detection of schistosomiasis japonica in a rabbit model.. Int J of Mass Spectrom.

[16] Yang H-Z, Cui B, Liu H-Z, Mi S, Yan J (2009). Blocking TLR2 Activity Attenuates Pulmonary Metastases of Tumor.. PLoS ONE.

[17] Xu N, Yao HP, Sun Z, Chen Z (2008). Toll-like receptor 7 and 9 expression in peripheral blood mononuclear cells from patients with chronic hepatitis B and related hepatocellular carcinoma.. Acta Pharmacol Sin.

[18] Orihara K, Nagata K, Hamasaki S, Oba R, Hirai H (2007). Time–course of Toll-like receptor 2 expression, as a predictor of recurrence in patients with bacterial infectious diseases.. Clin Exp Immunol.

[19] Hise AG, Daehnel K, Gillette-Ferguson I, Cho E, McGarry HF (2007). Innate immune responses to endosymbiotic Wolbachia bacteria in Brugia malayi and Onchocerca volvulus are dependent on TLR2, TLR6, MyD88, and Mal, but not TLR4, TRIF, or TRAM.. J Immunol.

[20] Kelly MG, Alvero AB, Chen R, Silasi DA, Abrahams VM (2006). TLR-4 signaling promotes tumor growth and paclitaxel chemoresistance in ovarian cancer.. Cancer Res.

[21] Hritz I, Mandrekar P, Velayudham A, Catalano D, Dolganiuc A (2008). The critical role of toll-like receptor (TLR) 4 in alcoholic liver disease is independent of the common TLR adapter MyD88.. Hepatology.

[22] Matzinger P (2002). The danger model: a renewed sense of self.. Science.

[23] Lien E, Sellati TJ, Yoshimura A, Flo TH, Rawadi G (1999). Toll-like receptor 2 functions as a pattern recognition receptor for diverse bacterial products.. J Biol Chem.

[24] Hayashi F, Smith KD, Ozinsky A, Hawn TR, Yi EC (2001). The innate immune response to bacterial flagellin is mediated by Toll-like receptor 5.. Nature.

[25] Motyka B, Reynolds JD (1991). Apoptosis is associated with the extensive B cell death in the sheep ileal Peyer’s patch and the chicken bursa of Fabricius: a possible role in B cell selection.. Eur J Immunol.

[26] Baccala R, Hoebe K, Kono DH, Beutler B, Theofilopoulos AN (2007). TLR-dependent and TLR-independent pathways of type I interferon induction in systemic autoimmunity.. Nat Med.

[27] Testro AG, Visvanathan K (2009). Toll-like receptors and their role in gastrointestinal disease.. J Gastroenterol Hepatol.

