# Supplementary figures and images for: Gene Silencing of Toll-Like Receptor 2 Inhibits Proliferation of Human Liver Cancer Cells and Secretion of Inflammatory Cytokines
Source: PLoS One. 2012 Jul 16;7(7):e38890. doi: 10.1371/journal.pone.0038890 (PMC3398009; doi:10.1371/journal.pone.0038890)

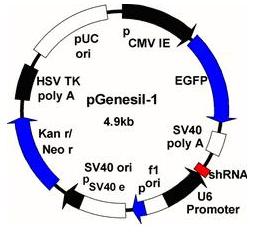

Supplement: Figure S1 — RNAi plasmid vectors (pGenesil-1 plasmid). (JPG) [file pone.0038890.s001.jpg]
